# Nursing protocols to reduce urinary tract infection caused by indwelling catheters: an integrative review

**DOI:** 10.1590/0034-7167-2022-0067

**Published:** 2023-03-06

**Authors:** Maria Estela de Queiroz Miranda, Marcelo Ricardo Rosa, Meire Cristina Novelli e Castro, Cassiana Mendes Bertoncello Fontes, Silvia Cristina Mangini Bocchi

**Affiliations:** I Universidade Estadual Paulista Júlio de Mesquita Filho. São Paulo, São Paulo, Brazil

**Keywords:** Urinary Catheterization, Catheter-Related Infections, Indwelling Catheters, Protocols, Nurses., Cateterismo Urinario, Infecciones Relacionadas con Catéteres, Catéteres de Permanencia, Evaluación en Enfermería, Enfermeras y Enfermeros., Cateterismo Urinário, Infecções Relacionadas a Cateter, Cateteres de Demora, Protocolos, Enfermeira e Enfermeiros.

## Abstract

**Objectives:**

to analyze the production of knowledge in research articles about the effectiveness of nursing protocols for reducing indwelling urinary catheter dwell time and catheter-associated urinary tract infection rate in hospitalized adult and older patients.

**Methods:**

an integrative review of three full articles, available in the MEDLINE Complete - EBSCO, Scopus and Web of Science databases, from 01/01/2015 to 04/26/2021.

**Results:**

the three protocols reduced infection rates, and from the review/synthesis of their knowledge, a level IV body of evidence emerged to compose the nursing care process aimed at reducing indwelling urinary catheter dwell time and catheter-associated urinary tract infection.

**Final Considerations:**

this process gathers scientific evidence to support the elaboration of nursing protocols and, consequently, the conduction of clinical trials on its effectiveness in reducing urinary tract infection by indwelling urinary catheter.

## RESUMO

**Objetivos:**

analisar a produção do conhecimento de artigos de pesquisas acerca da efetividade de protocolos de enfermagem para redução do tempo de permanência de sonda vesical de demora e da taxa de infecção do trato urinário relacionada ao cateter em pacientes adultos e idosos hospitalizados.

**Métodos:**

revisão integrativa de três artigos na íntegra, nas bases de dados MEDLINE *Complete* (EBSCO), Scopus e *Web of Science*, no período de 01/01/2015 a 26/04/2021.

**Resultados:**

os três protocolos reduziram as taxas de infecção, e, da revisão/síntese de seu conhecimento, emergiu um corpo de evidências de nível IV para compor o processo de cuidar de enfermagem, visando à redução da permanência do cateter e da infecção associada.

**Considerações Finais:**

esse processo reúne evidências científicas para subsidiar a elaboração de protocolos de enfermagem e, consequentemente, a condução de ensaios clínicos sobre sua eficácia na redução de infecção do trato urinário por sonda vesical de demora.

## RESUMEN

**Objetivos:**

analizar la producción de conocimiento a partir de artículos de investigación sobre la efectividad de los protocolos de enfermería para reducir la estancia de una sonda vesical y la tasa de infección del tracto urinario relacionada con el catéter en adultos y ancianos hospitalizados.

**Métodos:**

revisión integradora de tres artículos completos, en las bases de datos MEDLINE Complete (EBSCO), Scopus y Web of Science, del 01/01/2015 al 26/04/2021.

**Resultados:**

los tres protocolos redujeron las tasas de infección y, de la revisión/síntesis de sus conocimientos, surgió un cuerpo de evidencia de nivel IV para componer el proceso de atención de enfermería, con el objetivo de reducir la permanencia de sonda vesical y la infección asociada.

**Consideraciones Finales:**

este proceso reúne evidencias científicas para apoyar la elaboración de protocolos de enfermería y, consecuentemente, la realización de ensayos clínicos sobre su efectividad en la reducción de la infección por sonda vesical permanente.

## INTRODUCTION

In Brazilian hospital settings, bladder probing is one of the main invasive procedures that can lead to the development of healthcare-associated infections (IRAS)^(1)^. Within the nursing team and under medical prescription, nurses are responsible for catheter insertion and for care planning for patients with Indwelling Urinary Catheter (IUC), both procedures regulated by Resolution of the Federal Council of Nursing (COFEN) 0450/2013, aiming at prevention of Urinary Tract Infection (UTI), among other iatrogenic events arising from the device^(2-3)^.

According to COFEN Resolution 358/2009, Brazilian nurses do not have the autonomy to decide on the permanence or exchange of an IUC, unless the health institution has an operational protocol^(3)^. As in the Brazilian National Health Regulatory Agency (ANVISA - *Agência Nacional de Vigilância Sanitária*) recommendations, it is concluded that COFEN’s decision was based on an observational study carried out in hospitals in cities in the countryside of São Paulo, to assess nursing clinical practice with IUC insertion^(4)^. In this study, the authors considered that the procedure presents risks of trauma and urinary infection; therefore, due to its complexity, it requires medical prescription, material, person and process management^(5)^.

ANVISA recommends that urinary catheters should not be changed periodically, but only when: violation of the system and its contamination occur; presence of large amounts of residues or incrustations at the catheter tip; probe malfunction; system obstruction; and fever with no other recognized cause. ANVISA does not recommend regular bacteriological examinations, due to the high cost and the benefits not being as effective, however clinical observation should be rigorous^(4)^.

However, the literature points to indwelling catheter dwell as the most relevant risk factor for catheter-associated UTI, which can be modifiable^(6-7)^. Moreover, bacteriuria is a precursor of this type of infection, with a mean rate of 3% to 10% per day of catheterization so that all patients catheterized for one month will develop the infection^(6)^.

The main risk factors for UTI, related to the host and catheter, include: patients with comorbidities (diabetes mellitus and renal failure, serum creatinine > 2 mg/dL, at catheterization); females over 50 years of age; cases of catheter insertion after the 6^th^ day of hospitalization; increased bacterial colonization of the perineum; IUC insertion outside the operating room^(8-9)^.

Bloodstream infection associated with urinary catheter is rare event, occurring in less than 4% of cases^(10)^ and may be associated to males, immunosuppressive therapy, history of cancer, neutropenia, kidney disease, smoking in the last five years, and number of days in hospital before bacteriuria^(11)^. Thus, prevention strategies for patients at higher risk of bloodstream infection can also be incorporated into a general catheter-associated UTI prevention program.

It is emphasized that, in the United States, 70% of ITUs acquired in hospitals were due to urinary catheterization^(9)^, resulting in an increase in costs and hospitalization time by four days^(12)^; 65% to 70% of these are considered avoidable^(13)^.

The prevention of catheter-associated UTI has become a priority for most American hospitals, given the decision of two American health care programs (Medicare and Medicaid) to no longer reimburse hospitals for the extra costs of treating patients with acquired UTI^(14)^, due to the civil liability of these institutions.

Furthermore, since 2009, the Centers for Disease Control and Prevention (CDC), through the Guideline for Prevention of Catheter-Associated Urinary Tract Infections, have been considering guidelines and protocols for nurse-managed removal of urinary catheters as one of the strategies for the appropriate IUC use, aiming at to reducing the risk of UTI presented by these devices^(15)^.

Thus, in Brazil, the adoption of protocols for nurses to remove IUC from patients, in agreement with the medical team, can contribute to reducing catheter dwell and, consequently, UTI, hospital stay, costs with care and use of antibiotics.

## OBJECTIVES

To analyze the production of knowledge from research articles about the effectiveness of nursing protocols to reduce IUC dwell and catheter-associated UTI rate in hospitalized adult and older patients.

## METHODS

### Study design and research question

This is an integrative literature review, one of the research methods used in Evidence-Based Practice (EBP), as a resource for incorporating evidence into clinical practice, to gather and synthesize research results on a particular topic or issue, in a way that systematic and orderly^(16)^. The method comprised the six recommended steps: (1^st^) theme identification and hypothesis or research question selection; (2^nd^) establishment of criteria for inclusion and exclusion of studies/sampling or literature search; (3^rd^) definition of the information to be extracted from the selected studies/study categorization; (4^th^) assessment of studies included in the integrative review; (5^th^) interpretation of results; (6^th^) presentation of the review/synthesis of knowledge^(17)^.

To elaborate the research question, the PICO strategy^(18)^ was used, according to acronyms: P (population/patients) - hospitalized adults and older patients undergoing indwelling bladder catheterization (Foley tube); I (Intervention) - nursing protocol to reduce indwelling bladder catheter dwell time; C (Comparison/control) - IUC removal not implemented by protocol to shorten catheter dwell time; O (Outcome) - reduction of IUC dwell time and UTI rate. Satisfied, the research question was outlined: what is the effectiveness of nursing protocols to reduce IUC dwell time and UTI rate through the catheter in hospitalized adults and older people?

### Data source

The sample was selected by access to Latin American and Caribbean Literature on Health Sciences (LILACS), MEDLINE Complete (EBSCO), Scopus, Current Nursing and Allied Health Literature (CINAHL) and Web of Science (WoS) databases, without determining a specific search field (article title; abstract; keywords), but opting for “all fields”. The search strategy used was the controlled descriptors combined with Boolean operators, arranged in the Medical Subject Headings (MeSH). In LILACS, the Health Sciences Descriptors (DeSC) were used (Chart 1).

**Chart 1:** Boolean combination search strategies in the CINAHL, Scopus, Web of Science, MEDLINE complete (EBSCO), LILACS databases, from 01/01/2015 to 04/26/2021

Database	Boolean combinations
CINAHL with Full text (EBSCO)	protocol AND nurse-directed AND (urinary catheters or indwelling catheters or long-term catheters) AND (urinary tract infection or uti or tract infection or urinary infection)
Scopus	(ALL (protocol) AND ALL (“nurse-directed”) AND ALL (“urinary catheters” OR “indwelling catheters” OR “long-term catheters”) AND ALL (“urinary tract infection” OR uti OR “tract infection or urinary infection”)) AND ( LIMIT-TO ( PUBSTAGE, “final”)) AND (LIMIT-TO ( PUBYEAR, 2020) OR LIMIT-TO (PUBYEAR, 2019) OR LIMIT-TO (PUBYEAR, 2018) OR LIMIT-TO (PUBYEAR, 2017) OR LIMIT-TO (PUBYEAR, 2016) OR LIMIT-TO (PUBYEAR, 2015)) AND (LIMIT-TO (DOCTYPE, “ar”)) AND (LIMIT-TO (LANGUAGE, “English”) OR LIMIT-TO (LANGUAGE, “Spanish”) OR LIMIT-TO (LANGUAGE, “Portuguese”)) AND (LIMIT-TO (SRCTYPE, “j”))
Web of Science	(ALL=(nurse-directed AND protocol AND urinary catheters AND urinary tract infection)) Stipulated time: Last 5 years. Indices: SCI-EXPANDED, SSCI, A&HCI, CPCI-S, CPCI-SSH, ESCI, CCR-EXPANDED, IC.
MEDLINE complete (EBSCO)	protocol AND (urinary catheters or indwelling catheters or long-term catheters) AND (urinary tract infection or uti or tract infection or urinary infection) AND nursing
LILACS	*Cateterismo Urinário* OR *Cateterismo Uretral* AND *Enfermeiras e Enfermeiros*

We included complete articles with abstracts and that answered the research question, in Portuguese, English and Spanish, published in national and international journals, indexed in the aforementioned databases, from 01/01/2015 to 04/26/2021. This period, close to five years, was selected to seize updated protocols.

**Figure 1:**
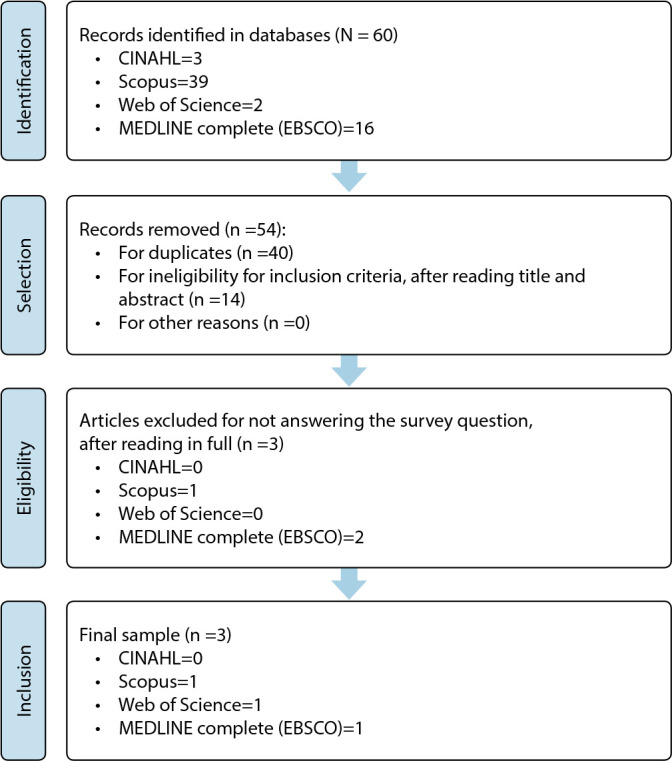
Sample constitution flowchart, adapted from PRISMA^(19)^

All records resulting from the databases (60) were organized, by Mendeley reference manager, into folders named with the databases from which the articles took place. This procedure made it possible to eliminate duplicates (40), as well as, after reading title and abstract, those that did not meet the inclusion criteria (14). Thus, the eligible ones were obtained, thus forming a corpus of analysis consisting of six articles, which were read in full. The reading also made it possible to exclude three (3) that did not respond to the review question. Thus, the final sample was held to three articles (Figure 1).

### Data collection and organization

For analysis of the corpus of articles, instruments were used that made up Tables 2 and 3, with the following data extracted: authors’ surname; year; periodical; country; scenario where the study took place; article language; objectives; protocol, main results/outcomes; and research conclusions. Articles that met the inclusion criteria were independently translated and assessed by two members of the research team, for later consensus on inclusion, translation and the extracted data. Disagreements were investigated by a third researcher.

**Chart 2:** Characterization of research articles with the outcome variable “catheter-associated Urinary Tract Infection rate”, having as the exposure variable “nursing protocol to reduce catheter dwell time” according to author(s), year of publication, country and scenarios where the studies took place, as well as study design, level of evidence and objective(s), published in journals indexed in Scopus, Web of Science and MEDLINE databases complete (EBSCO), from 01/01/2015 to 04/26/2021

Authors/year/journal/database	Country/setting/language of article	Study design/level of evidence	Objective(s)
Article 1 (Tyson et al., 2020)^(23)^/Implementation of a nurse-driven protocol for catheter removal to decrease catheter-associated urinary tract infection rate in a surgical trauma ICU/Journal of intensive care medicine/Web of Science.	United States of America/Surgical ICU of a large trauma care center - Carolinas Medical Center/English	Retrospective cohort study/IV	Compare the catheter-associated Urinary Tract Infection rate before and after implementation of a urinary catheter removal protocol by a nurse in a trauma surgery ICU.
Article 2 (Major-Joynes, Pegues, Bradway, 2016)^(24)^/A Nurse-driven protocol for removal of indwelling urinary catheters across a multi-hospital academic healthcare system/Urologic nursing/MEDLINE complete (EBSCO)	United States of America - Pennsylvania/ICUs of three teaching hospitals/English	Retrospective cohort study/IV	Implement a nurse IUC removal protocol at three American teaching hospitals to reduce catheter use and catheter-associated UTI rates.
Article 3 (Thomas, 2016)^(25)^/Reduction of catheter-Associated urinary tract infections through the use of an evidence-based nursing algorithm and the implementation of shift nursing rounds/Journal of Wound, Ostomy and Continence Nursing/SCOPUS.	United States of America/University Hospital - Coronary ICU with 28 beds/English	Quasi-experimental case-control study/IV	General: reduce the catheter-associated UTI rate in the ICU. Specific: decrease the rate of this type of infection, as well as catheter use in days; assess protocol use and compliance, involving nursing assessments of IUC, performed three times a day (10:00, 16:00 and 22:00) and a catheter removal algorithm conducted by a nurse.

It is noteworthy that, during article analysis, they were classified according to the levels of evidence proposed by Melnyk and Fineout-Overholt^(20)^, with the quality analyzed according to the tools provided in EQUATOR^(21)^.

The seven levels to qualify scientific evidence, according to Melnyk and Fineout Overholt^(20)^, are: level I: evidence from a systematic review or meta-analysis of all randomized controlled trials or from clinical guidelines based on systematic reviews of randomized controlled trials; level II: evidence derived from well-designed randomized controlled clinical trials; level III: evidence obtained from well-designed clinical trials without randomization; level IV: evidence from well-designed cohort and case-control studies; level V: evidence from a systematic review of qualitative and descriptive studies; level VI: evidence derived from a single descriptive or qualitative study; level VII: evidence from the opinion of authorities and/or expert committees report.

### Ethical aspects

As this is review research, carried out exclusively with scientific articles that respect national and international ethical principles, this study was exempt from records and assessments by the REC/CONEP system, as provided in Resolution 510 of 04/07/2016, Art. 1, sole paragraph, item VI^(22)^.

## RESULTS

The three nursing protocols proved to be effective in reducing IUC dwell time and, consequently, UTI rate, in hospitalized adult and older patients.

These protocols were assessed through primary, quantitative research, designed from observational studies, with two cohort studies, one published in 2020^(23)^ and the other in 2016^(24)^, and a case-control study published in 2016^(25)^. These provide level “IV” evidence, as they are well-designed studies, according to the Strengthening the Reporting of Observational Studies in Epidemiology (STROBE) Statement: guidelines for reporting observational studies^(26)^.

Furthermore, they are articles published in English, in journals with an International Standard Serial Number (ISSN) and indexed in international databases, such as Scopus, Web of Science, or MEDLINE complete (EBSCO). All conducted in Intensive Care Units in the United States of America.

In the first^(23)^, there was a reduction from 5.1 to 2.0 infections per 1,000 catheter-days in the protocol pre- and post-implantation period, while in the second^(24)^, this reduction was 19 %. For the authors, the success was not greater because the study was carried out in three hospitals, and in one of them, nurses did not comply with the protocol. However, in the other, although nurses were attentive to the protocol, there was also no reduction.

However, in the third article^(25)^, in which there is a mean rate of 91% of nurses’ compliance with the protocol, nine months after implantation, there was a reduction in the median of catheter-associated UTI from 10.31 to 0.00.

In Charts 2 and 3, there is the individual synthesis of the articles that made up the analysis corpus, contributing to the interpretation of results.

**Chart 3:** Main results and conclusions of articles derived from research that adopted as an outcome variable the “catheter-associated Urinary Tract Infection rate”, associated with the exposure variable “protocol managed by nurses to reduce catheter dwell time”, published in journals indexed in Scopus, Web of Science and MEDLINE complete (EBSCO) databases, from 01/01/2015 to 04/26/2021

Authors	Protocol/bundle	Results/ outcomes and conclusions
(Article 1) Tyson et al. (2020)^(23)^	**The nursing intervention to reduce UTI by IUC is characterized in a bundle structured in three instruments managed by nurses (A, B and C):** **A. Care and maintenance of Long-Term IUC** 1. Perform hand hygiene immediately before and after any handling of the catheter or associated equipment; 2. Keep the internal catheter properly protected, with stabilization device to avoid movement and traction/urethral trauma; 3. Keep the drainage system continuously closed; 4. Do not disconnect catheter and drainage tubes unless irrigation is required or drainage bag leakage occurs; 5. Empty the drainage bag regularly to prevent overflow, taking care that the faucet does not touch the sides of the urine collection container; 6. Keep a clean and labeled urine collection container for each patient, rinsing it with running water and storing it in a way to facilitate drying after each use; 7. Keep the catheter and drainage tube patency for the bag without folds; 8. Keep the urinary drainage bag below bladder level permanently; 9. Prevent the urine-collecting bag from touching the floor; 10. Avoid catheter exchanges at arbitrary fixed intervals; 11. Change the urinary catheter only with clinical indications, such as infection, obstruction, leakage or kidney/ureteral stones; 12. Sanitize the urinary meatus with soap and water daily and as needed, as in the case of fecal incontinence; 13. Replace catheter and urinary bag if accidentally disconnected, i.e., with compromised closed system.	**Resultados/desfechos** Catheter utilization decreased significantly with the implementation of the protocol conducted by nurses, from 0.78 in the pre-intervention period to 0.70 in the post-intervention period (p < 0.05). As a result of the bundle, the catheter-associated UTI rate decreased significantly, from 5.1 to 2.0 infections per 1,000 catheter-days, in the pre- and post-implementation period (Incident rate [IRR]: 0.38, 95% confidence interval. **Conclusions** Implementing a protocol/bundle for early IUC removal by nurses, as part of a multimodal intervention strategy for UTI, can result in measurable measures of reduction, both in the period of catheter use, and in the rates of this type of infection.
(Article 1) Tyson et al. (2020)^(23)^	**B. Flowchart for decision-making in IUC daily assessment** 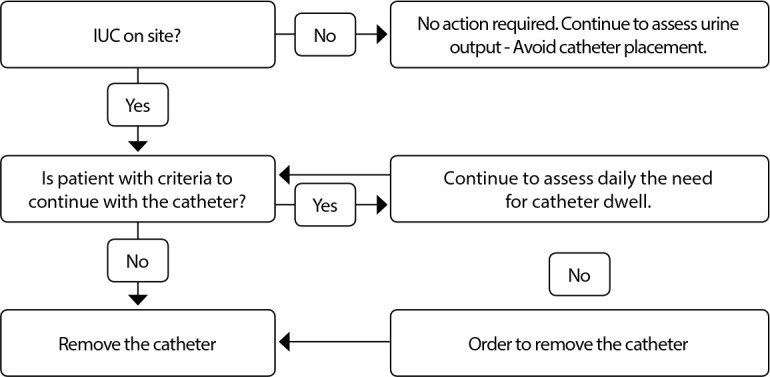 **C. Indications for continuous IUC use** 1. Patients with a urinary catheter passed by a urologist; 2. Urologist as responsible physician of a patient or this awaiting consultation with a specialist; 3. In the immediate postoperative period (not more than 24 hours); 4. Bladder outlet obstruction; 5. Urological/perineal procedures; 6. Continuous bladder irrigation; 7. Movement intolerance due to severe disability (severe contractures, pelvic or hip fractures) 8. On-site epidural; 9. Deep sacral/gluteal tissue injury and incontinent; 10. Stage III/IV/Unstable sacral/perineal pressure ulcer and incontinent; 11. Critically ill patients requiring urine output monitoring every 1 to 2 hours; 12. Patients chemically paralyzed or seeded and on mechanical ventilation; 13. Prolonged deep sedation (> 2 hours); 14. Comfort care at the end of life; 15. Urinary retention: Bladder with residual volume > 100 mL of urine after urination; Bladder with residual volume > 300 mL of urine at any time; Intermittent catheterization in 2 weeks, with volume > 300 mL of urine.	
Article 2 (Major-Joynes, Pegues, Bradway, 2016)^(24)^/A nurse-driven protocol for removal of indwelling urinary catheters across a multi-hospital academic healthcare system/ Urologic nursing/ MEDLINE	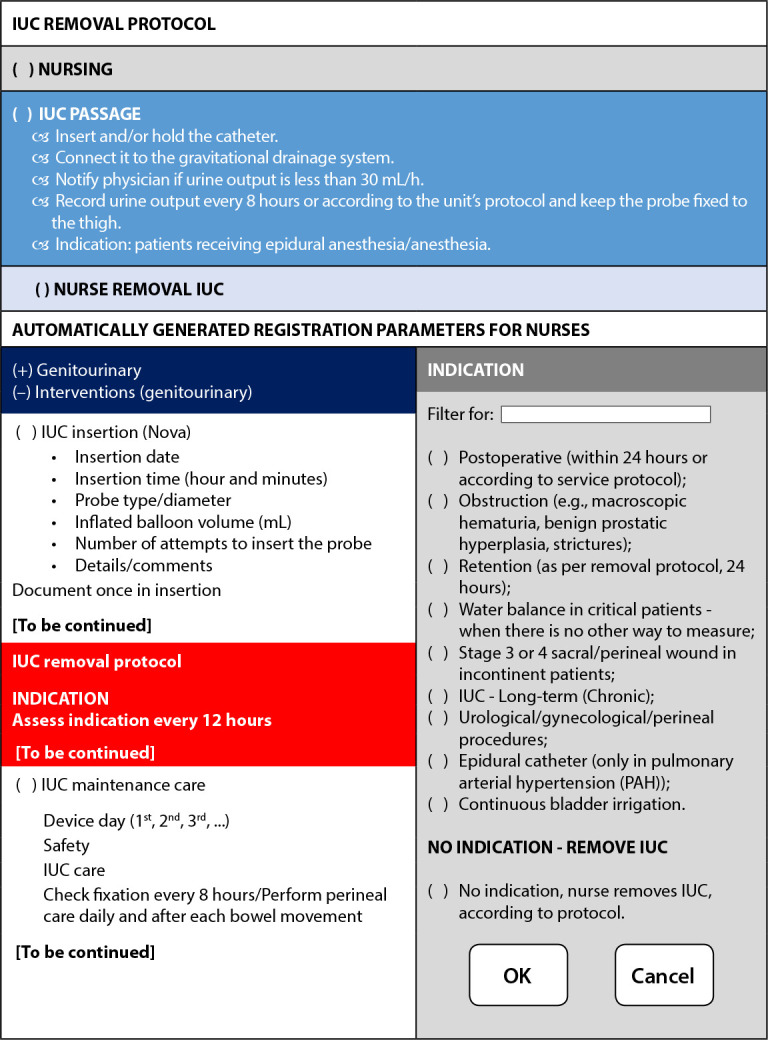	**Results/outcomes** With the adoption of the protocol, considering the ICUs of the three hospitals, there was a 19% reduction in UTI rates related to urinary catheterization per 1,000 catheterization days, compared to the baseline period (p = 0.13). However, the impacts on hospitals were different when analyzed separately. In Hospital 1, it was 28%, and it was associated with team compliance with the protocol, but in Hospital 2 the infection rate increased, despite the fact that the team used the protocol, while, in Hospital 3, it remained unchanged, but justified by low compliance of participants. **Conclusions** The project’s success included: 1) a cohesive multidisciplinary team (nursing, medical, information systems, education, statisticians, among others); 2) executive leadership support; and 3) engagement of medical, nursing and other teams involved in patient care in the units. To expand the initiative, it is necessary to explore the barriers to early adoption of an IUC Removal Protocol managed by nurses.
Article 3 (Thomas, 2016)^(25)^/ Reduction of catheter-associated urinary tract infections through the use of an evidence-based nursing algorithm and the implementation of shift nursing rounds/ Journal of Wound, Ostomy and Continence Nursing/Scopus	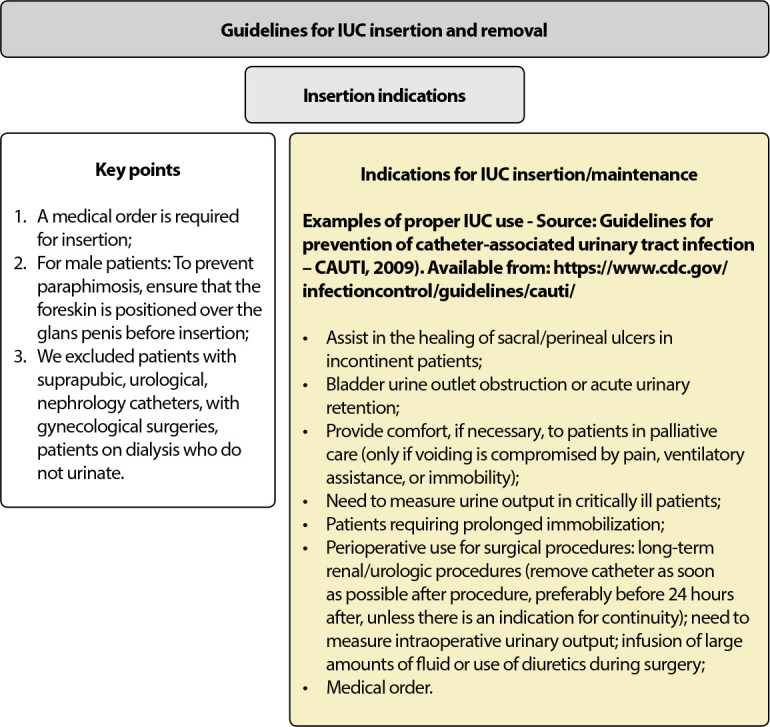	**Results/outcomes** There was a statistically significant change in the number of UTIs by IUC (p = 0.009) and occurrences (p = 0.005) after the intervention. The pre-intervention median went from 10.31 to 0.00 after nine months. The number of days of indwelling catheter and its use did not differ significantly after implementation. Nurses’ compliance with the intervention was calculated for each month, with a mean rate of 91%. The results indicate that an evidence-based practice project, managed by nurses, positively influenced the reduction of UTI by IUC. **Conclusions** The nursing protocol promoted evidence-based, nurse-led practice change to reduce catheter-associated UTI. Success was due to the change in nursing care culture.
Article 3 (Thomas, 2016)^(25)^/ Reduction of catheter-associated urinary tract infections through the use of an evidence-based nursing algorithm and the implementation of shift nursing rounds/Journal of Wound, Ostomy and Continence Nursing/Scopus	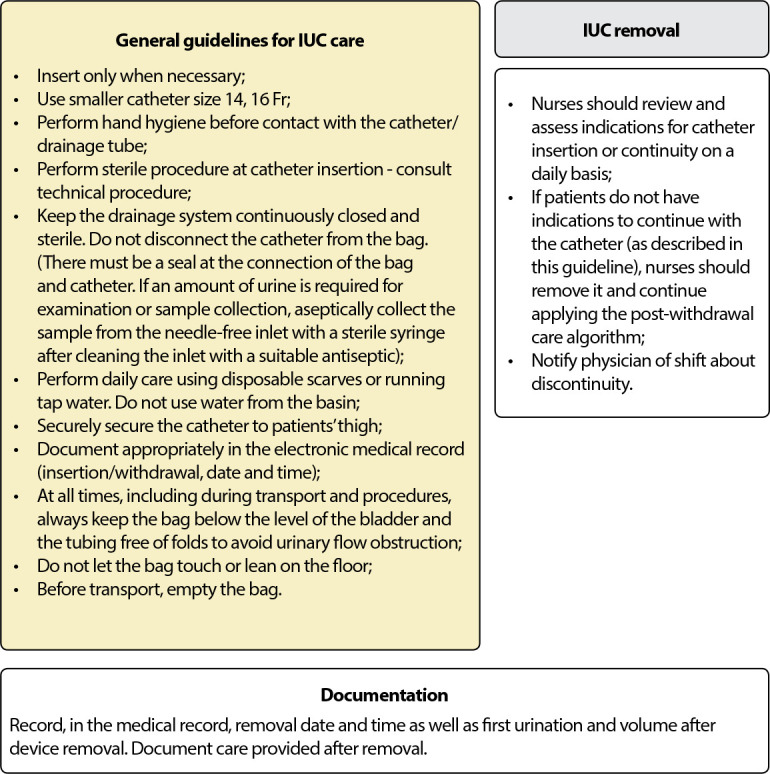	
Article 3 (Thomas, 2016)^(25)^/ Reduction of catheter-associated urinary tract infections through the use of an evidence-based nursing algorithm and the implementation of shift nursing rounds/ Journal of Wound, Ostomy and Continence Nursing/ Scopus	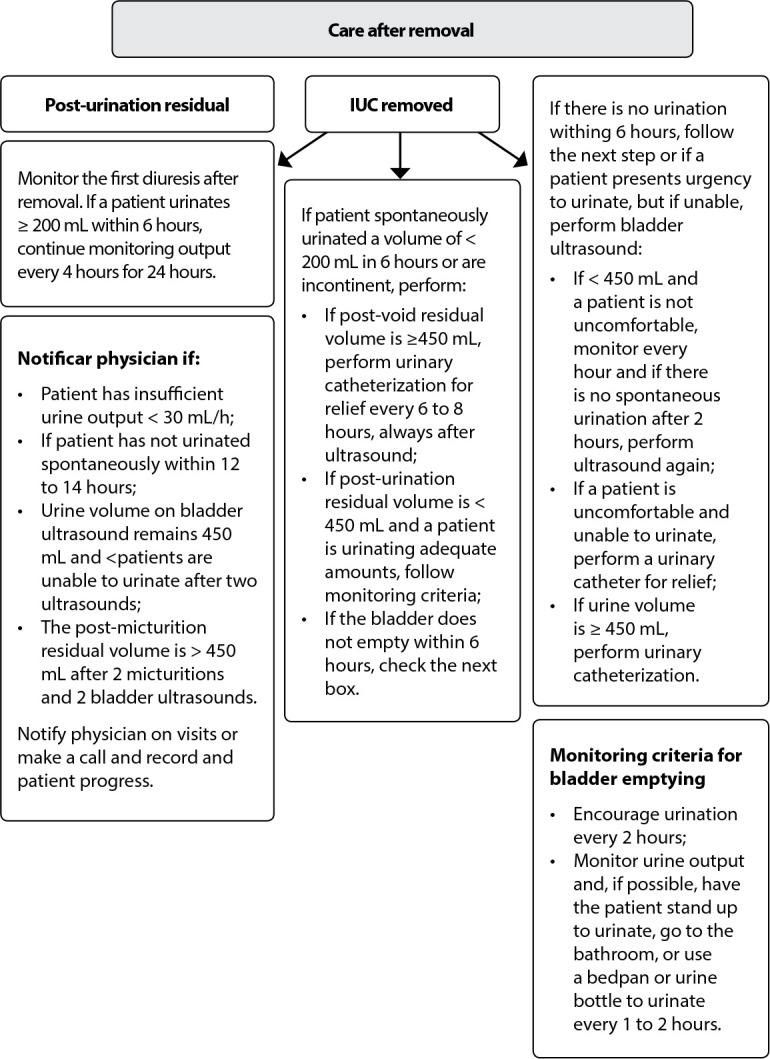	

## DISCUSSION

**Figure 2:**
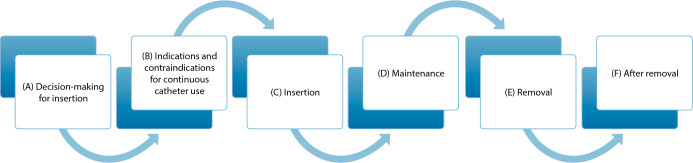
Diagram - Nursing care process to reduce Indwelling Bladder Tube and catheter-associated Urinary Tract Infection dwell time in adult and older patients. Review/synthesis of knowledge resulting from articles published in journals indexed in Scopus, Web of Science and MEDLINE complete (EBSCO) databases, from 01/01/2015 to 04/26/2021

Considering the effectiveness of the three nursing protocols to reduce IUC dwell and, consequently, UTI rates, the 6^th^ and last stage of the integrative review method was carried out, with presentation of review/synthesis of knowledge learned and represented in Figure 2.

### (A) Decision-making for insertion

Decision-making for IUC insertion is carried out upon medical prescription, taking into account criteria for inclusion^(25)^ and exclusion of patients from the procedure, including recommending using a flowchart for decision-making based, initially, on urinary output assessment for its installation^(23)^.

### (B) Indications and contraindications for continuous catheter use

IUC use is indicated for patients: (i) with a urinary catheter inserted by a urologist - the physician in charge is a urologist - or when patient is awaiting consultation with a specialist; (ii) in the immediate postoperative period (not longer than 24 hours)^(23)^ or according to the service protocol^(24)^; with bladder outlet obstruction^(23)^, such as macroscopic hematuria, benign prostatic hyperplasia and strictures^(24)^; with urological/gynecological/perineal procedures; (iii) with continuous bladder irrigation^(23-24)^; (iv) with movement intolerance, due to severe disability, such as severe contractures, pelvic or hip fractures^(23)^; (v) receiving epidural anesthesia/anesthesia; (vi) incontinent patients with stage III and IV pressure ulcers in the sacral, perineal and gluteal regions; (vii) critical need to monitor urinary output every 1 to 2 hours (water balance), when there is no other way to measure^(23-24)^; (viii) under prolonged deep sedation and/or mechanical ventilation or (> 2 hours); (ix) with comfort care at the end of life; (x) with urinary retention(23), according to the 24-hour removal protocol^(23)^: (a) bladder with residual volume > 100 mL of urine after urination; (b) bladder with residual volume > 300 mL of urine at any given time; (c) intermittent catheterization in two weeks, with volume > 300 mL of urine^(23)^; (d) with indication of long-term (chronic) IUC^(24)^.

It is noteworthy that IUC insertion by nurses is contraindicated in patients under the care of medical specialties, such as urology and nephrology, and patients on dialysis who do not urinate, as well as in those with gynecological surgeries or who are using suprapubic catheters (cystostomies)^(25)^.

### (C) Catheter insertion

Care with IUC insertion involves: using a smaller 14, 16 Fr foley tube; making sure that the technical procedure is aseptic; preventing paraphimosis before catheter insertion in male patients so that the foreskin is positioned over the glans penis^(25)^; inserting and/or maintaining the catheter connecting to the gravitational drainage system; securing the catheter to patients’ thigh; notify physician if urine output is less than 30 mL/h^(24)^; record, in the medical record, the exact date and time (hours and minutes)^(24-25)^, probe type and gauge, balloon inflated volume (mL), number of attempts to insert the probe, details/comments^(24)^.

### (D) Catheter maintenance

After inserting the IUC, the protocols propose maintenance care, recommending: hand hygiene before and after any handling of the catheter, drainage tube and/or collection bag^(23,25)^; keep the internal catheter properly stabilized by fixation on patients’ thigh, avoiding movement and urethral traction/trauma; keep the drainage system continuously closed/sterile^(23)^, so as not to disconnect the catheter from the drainage system to the collection bag, including a seal in the connection between the catheter and the drainage system, preventing it from breaking; if it is necessary to collect urine for exams, do it aseptically from the inlet and without a needle, with a sterile syringe, after cleaning the inlet with a suitable antiseptic^(25)^; do not disconnect the catheter from the drainage system unless irrigation is required or leakage from the drainage bag occurs; empty the drainage bag regularly to prevent overflow, taking care that the faucet does not touch the sides of the urine collection container; keep a clean and labeled urine collection container for each patient, rinsing it with running water and storing it in a way that facilitates drying after each use; keep the catheter and drainage tube pervious to the bag and no folds^(23)^; keeping the urinary drainage bag below the level of the bladder permanently, preventing it from touching or leaning on the floor^(23,25)^; avoid catheter exchanges at arbitrary fixed intervals, but only for situations of clinical indications, such as infection, obstruction, leakage or renal/ureteral stones; replace catheter and urinary bag if accidentally disconnected, i.e., with compromised closed system^(23)^; record urine production every 8 hours or according to the unit’s protocol, keeping the probe fixed to the thigh, as well as the day of the device (1^st^, 2^nd^, 3^rd^, ...)^(24)^; keep the collection bag permanently, including during transport and procedures, always below the level of the bladder and tubing free of folds to avoid urinary flow obstruction; empty the collection bag before transporting patients^(25)^.

It is noteworthy that, among the items that make up the care with the IUC maintenance, the three protocols mentioned the need to adopt care related to perineal hygiene daily and after bowel movements. However, while article 2^(24)^ did not specify the products to be used in the procedure, articles 1 and 3 disagreed with them. In the protocol presented by article 1, it is recommended to sanitize with water and soap daily and as needed, as in the case of fecal incontinence^(23)^, while in the protocol of article 3, it is proposed to perform daily care with disposable tissues or running water, without using water from the bathing basin^(25)^.

Fortunately, results of a quasi-experimental study showed that washing the perineal region every 12 hours with 2% chlorhexidine solution reduced the incidence of UTI (13.3%), compared to that of women admitted to the ICU, submitted to the same type of hygiene, but with saline solution (76.7%)^(27)^. Further studies are needed to assess the effectiveness of products, such as soaps sold for intimate hygiene, which are dermatologically tested, with balanced pH and, therefore, helping to preserve natural defenses.

### (E) Catheter removal

For catheter removal, it is recommended to use a flowchart in daily assessment of indications of whether or not to maintain the IUC, as shown in Chart 3 ^(23)^. If there is no indication, nurses should remove it and use the care algorithm after removal, recording them in the medical record, including the exact date and time of removal and, finally, notifying physician about the procedure^(25)^.

In the IUC removal phase, specifically in the time interval in which the nurse should reassess the indication for urinary catheter maintenance, of the three protocols, one recommends every 12 hours^(25)^ and the other two, daily^(23,25)^. Analyzing this time interval and the impact on catheter-associated UTI, in the three protocols, it was found that all had an impact, however those performed daily were higher^(23,25)^.

### (F) After catheter removal

For care after IUC removal: use monitoring criteria for bladder emptying, such as encouraging urination every 2 hours, monitoring urine output, if possible, having patient stand up to urinate, go to the bathroom, or using a bedpan or urine bottle to urinate every 1 to 2 hours and assessing the first diuresis after catheter removal. If patient urinates ≥ 200 mL within 6 hours, continue monitoring output every 4 hours for 24 hours. Notify physician if: (a) patient has insufficient urinary output (< 30mL/h); (b) patient does not urinate spontaneously within 12 to 14 hours; (c) urinary volume on bladder ultrasound (US) remains < 450 mL and patient is unable to urinate after two US; (d) residual volume after voiding is > 450 mL after 2 voids and 2 bladder US. Perform US if patient spontaneously urinates < 200 mL within 6 hours or is incontinent: (a) if post-void residual volume is ≥ 450 mL, perform urinary catheterization for relief every 6 to 8 hours, always after US; (b) if post-micturition residual volume is < 450 mL and patient is urinating adequate amounts, follow monitoring criteria. If the bladder does not empty within 6 hours, the frequency of urinary catheterization for relief is determined by comfort and maintaining a total urine volume in the bladder < 450 mL. It should be performed at most twice every 6 to 8 hours. If there is no urination within 6 hours, follow the next steps or if patient has an urge to urinate but is unable to perform bladder US: (a) if < 450 mL and patient is not uncomfortable, monitor hourly, and if not present spontaneous urination after 2 hours, perform US again; (b) if patient is uncomfortable and unable to urinate, perform a urinary catheter for relief; (c) if urine volume is ≥ 450 mL, perform urinary catheterization for relief. Notify physician during visits or by phone and record patient progress^(25)^.

It is verified that the care after IUC removal will demand nurses with competence in US use, to estimate the bladder residual volume, which refers to the need to include the formation of this competence in the curricula of undergraduate nursing, once COFEN Resolution 679/2021 approved the regulation of US at the bedside and in the pre-hospital environment as a private activity of nurses, within the scope of the nursing team, through specific training^(28)^.

### Study limitations

There was a scarcity of research. All three were carried out in the same country, the United States of America, and in ICUs, i.e., in closed and controlled environments, as well as using methodological designs that produced level IV scientific evidence. These facts made it impossible to compare whether the effectiveness would be the same when the object of investigation was conducted in other scenarios, as well as through randomized and controlled clinical trials, to assess the protocol effectiveness.

### Contributions to nursing

This is a relevant thematic study, with contributions that go beyond the exercise of professional autonomy, also dealing with patient safety defense, civil liability of health institutions and non-multi-resistance of hospital microorganisms. It signals to nurses, as well as to undergraduate courses, the need to establish competence for US use at the bedside, especially in the phase after IUC removal.

## FINAL CONSIDERATIONS

Nursing protocols have been shown to be effective in reducing IUC dwell time and UTI rate in adult and elderly patients hospitalized in ICUs in the United States, reaching zero this type of infection, when the mean nurses’ compliance with the protocol was greater than 90%. These protocols were assessed through observational studies, with levels of evidence of level IV.

The review/synthesis of results, the last step of the integrative review method, allowed proposing the nursing care process to reduce IUC dwell time and catheter-associated UTI, in adult and older patients, according to the stages: (a) decision-making for insertion; (b) indications and contraindications for continuous catheter use; (c) insertion; (d) maintenance; (e) removal; (f) after removal.
